# Supplementary figures and images for: Genetic Investigation of Consanguineous Pakistani Families Segregating Rare Spinocerebellar Disorders
Source: Genes (Basel). 2023 Jul 6;14(7):1404. doi: 10.3390/genes14071404 (PMC10379343; doi:10.3390/genes14071404)

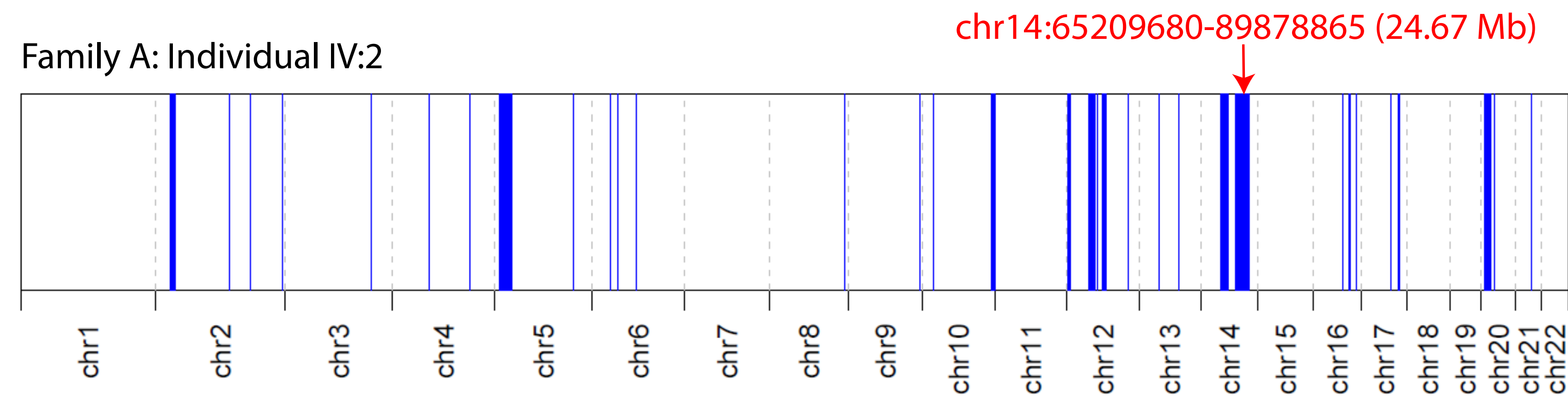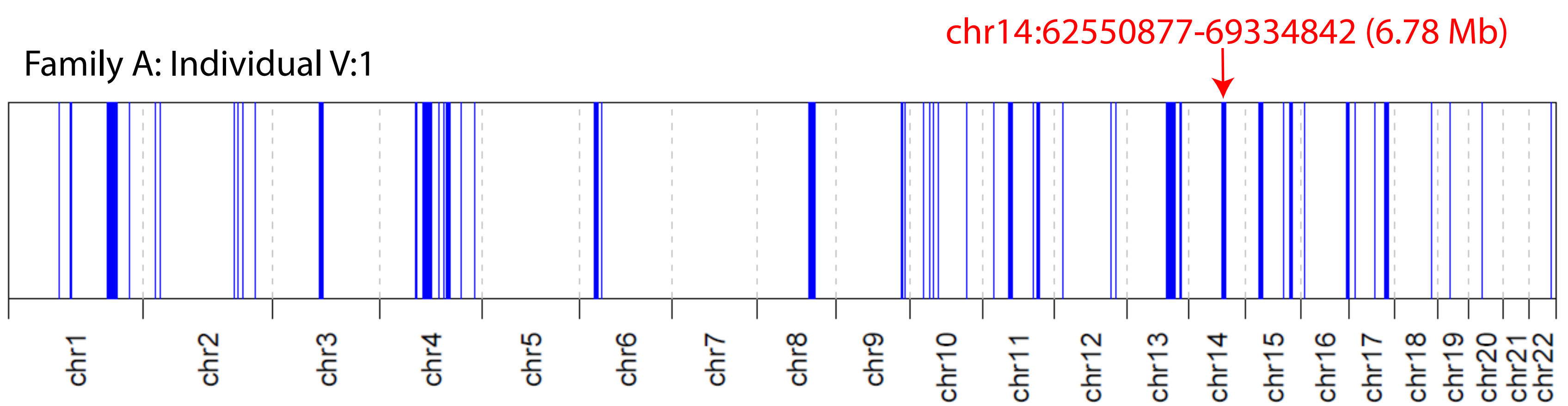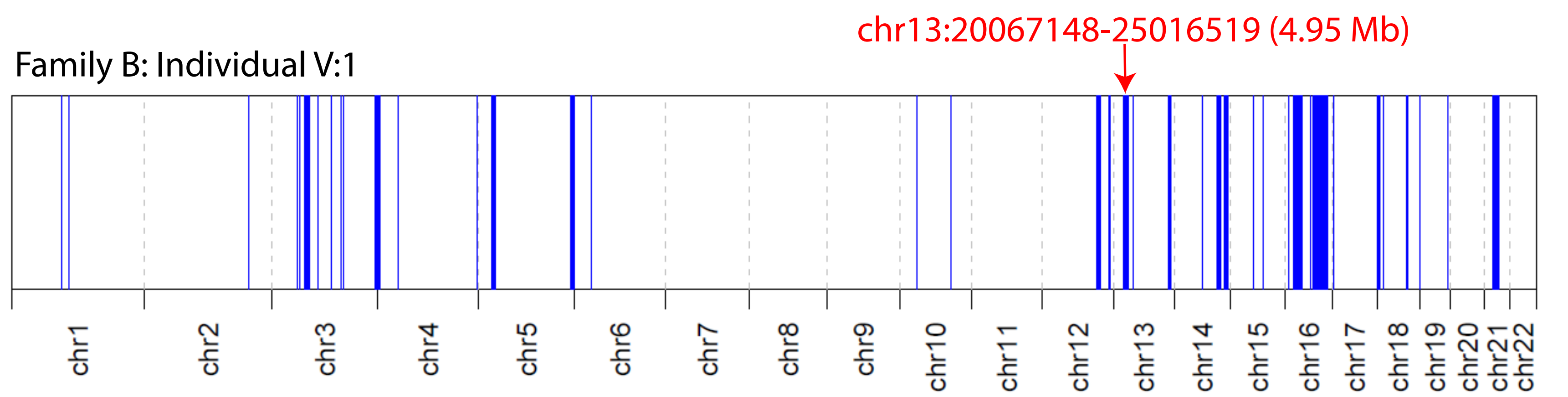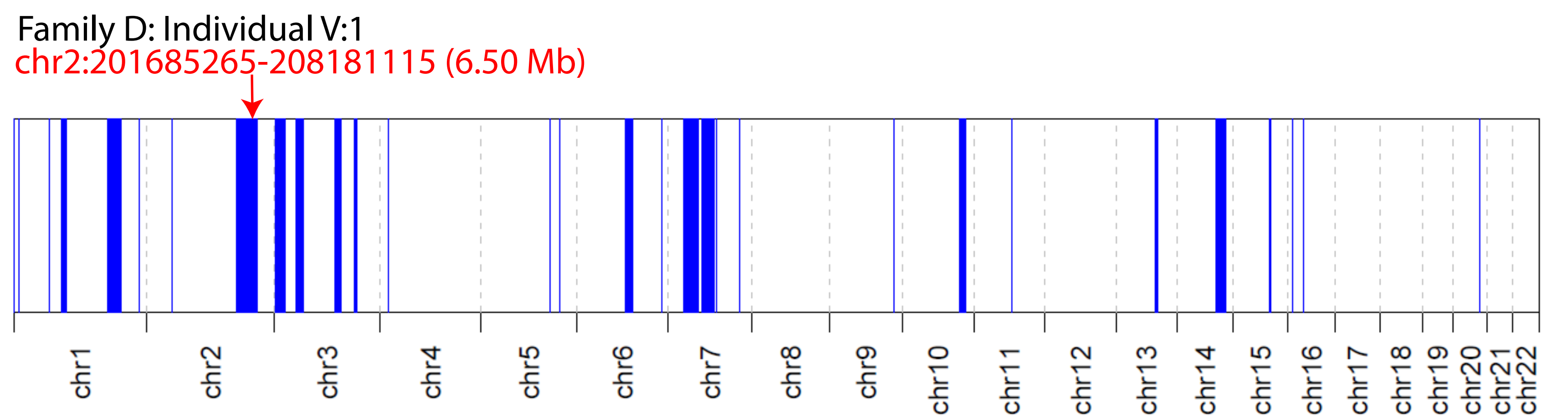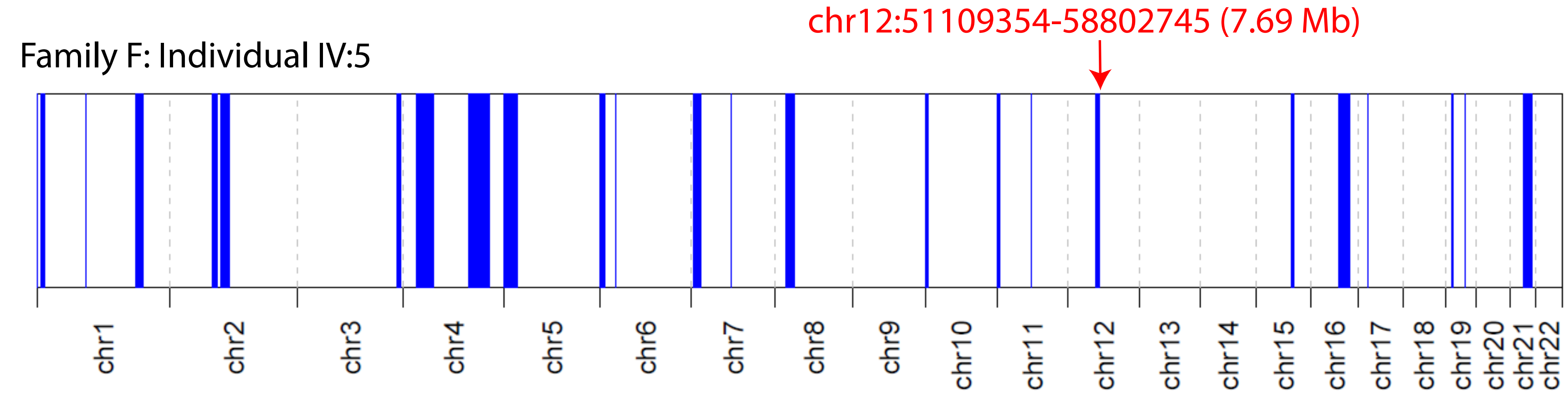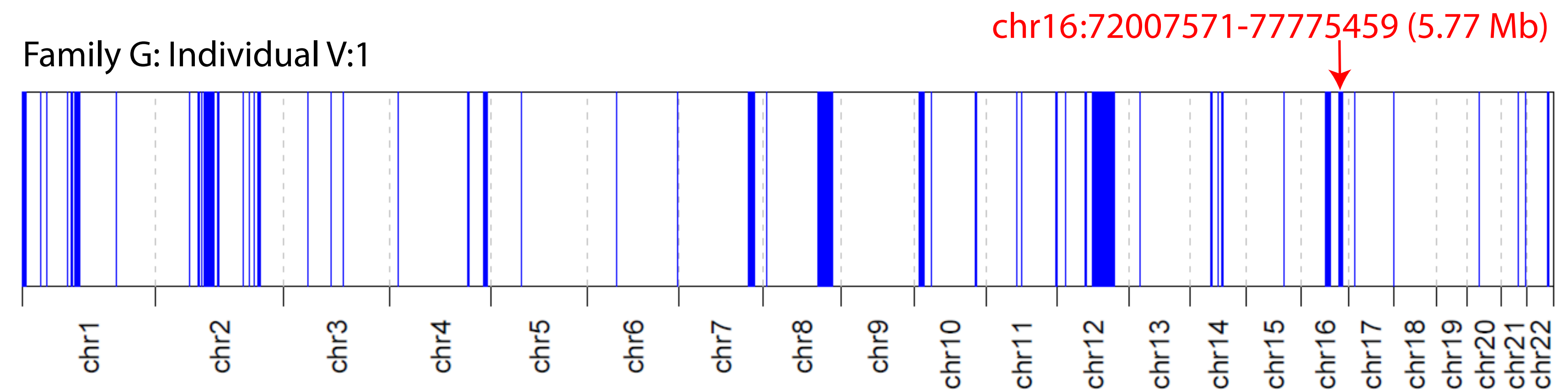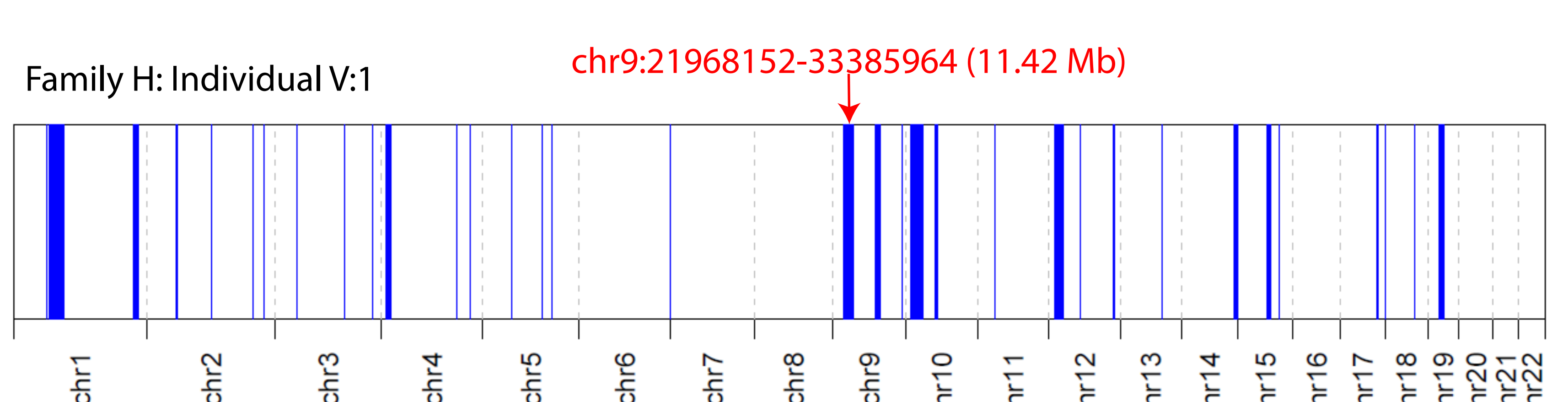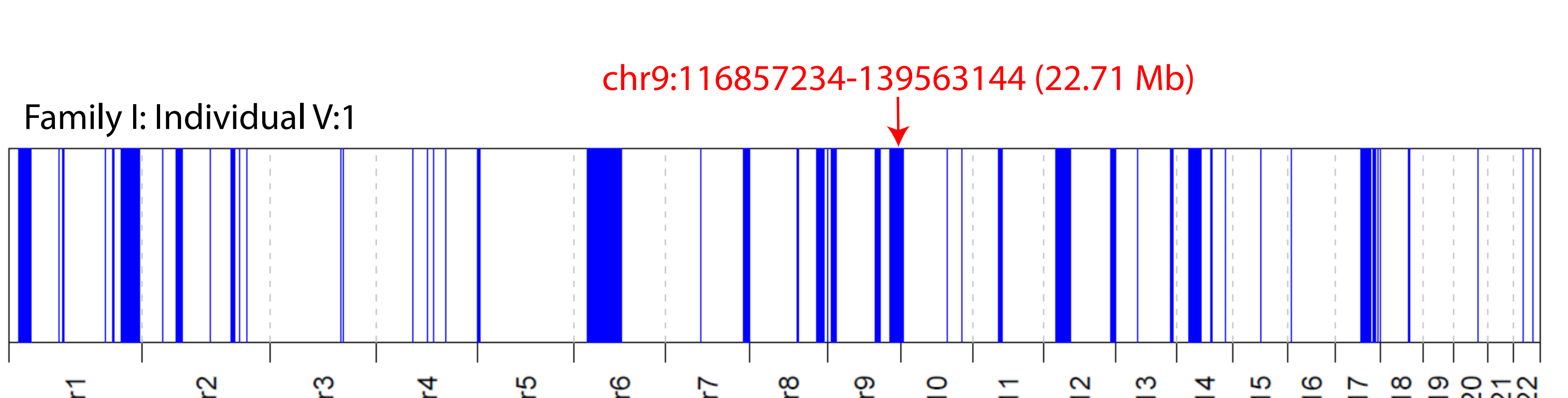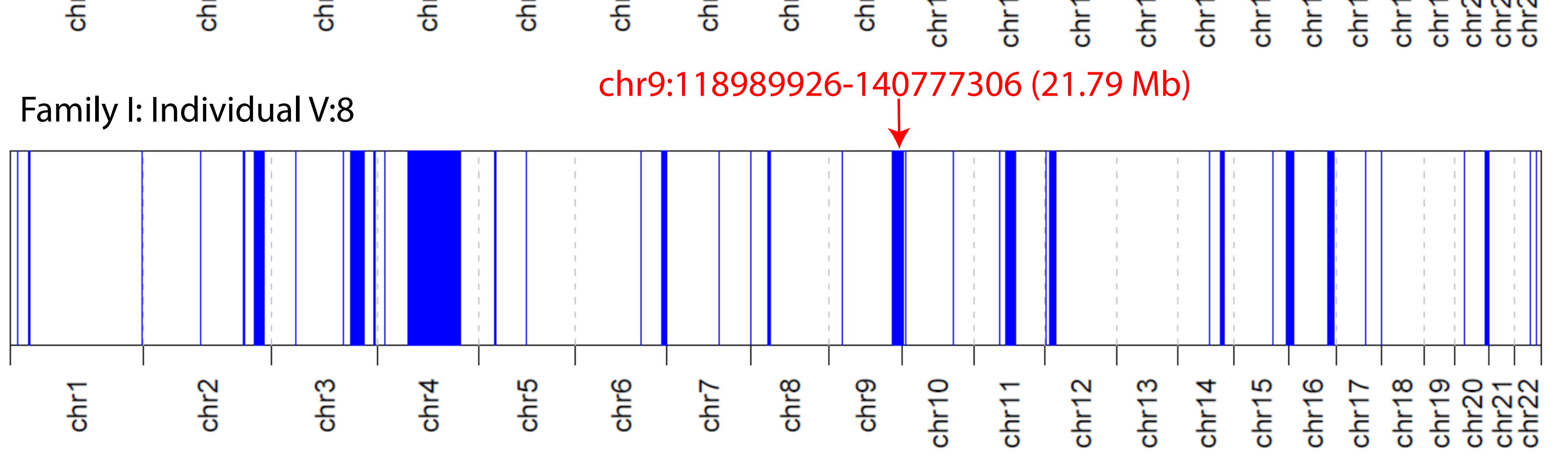

Supplement: Supplementary file 1 [file genes-14-01404-s001.zip › Figure S1..pdf]

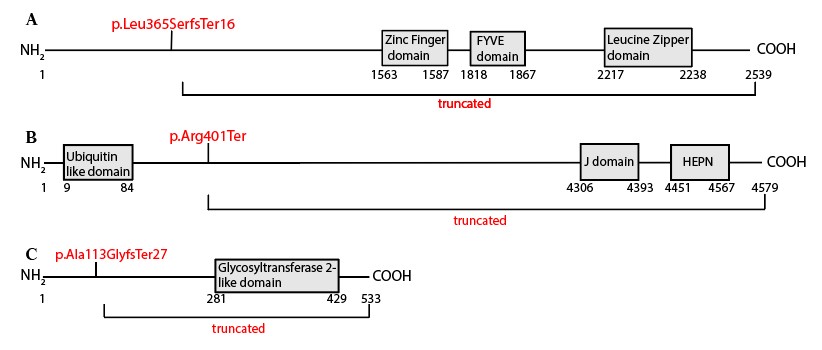

Supplement: Supplementary file 1 [file genes-14-01404-s001.zip › Figure S2..jpg]

**A**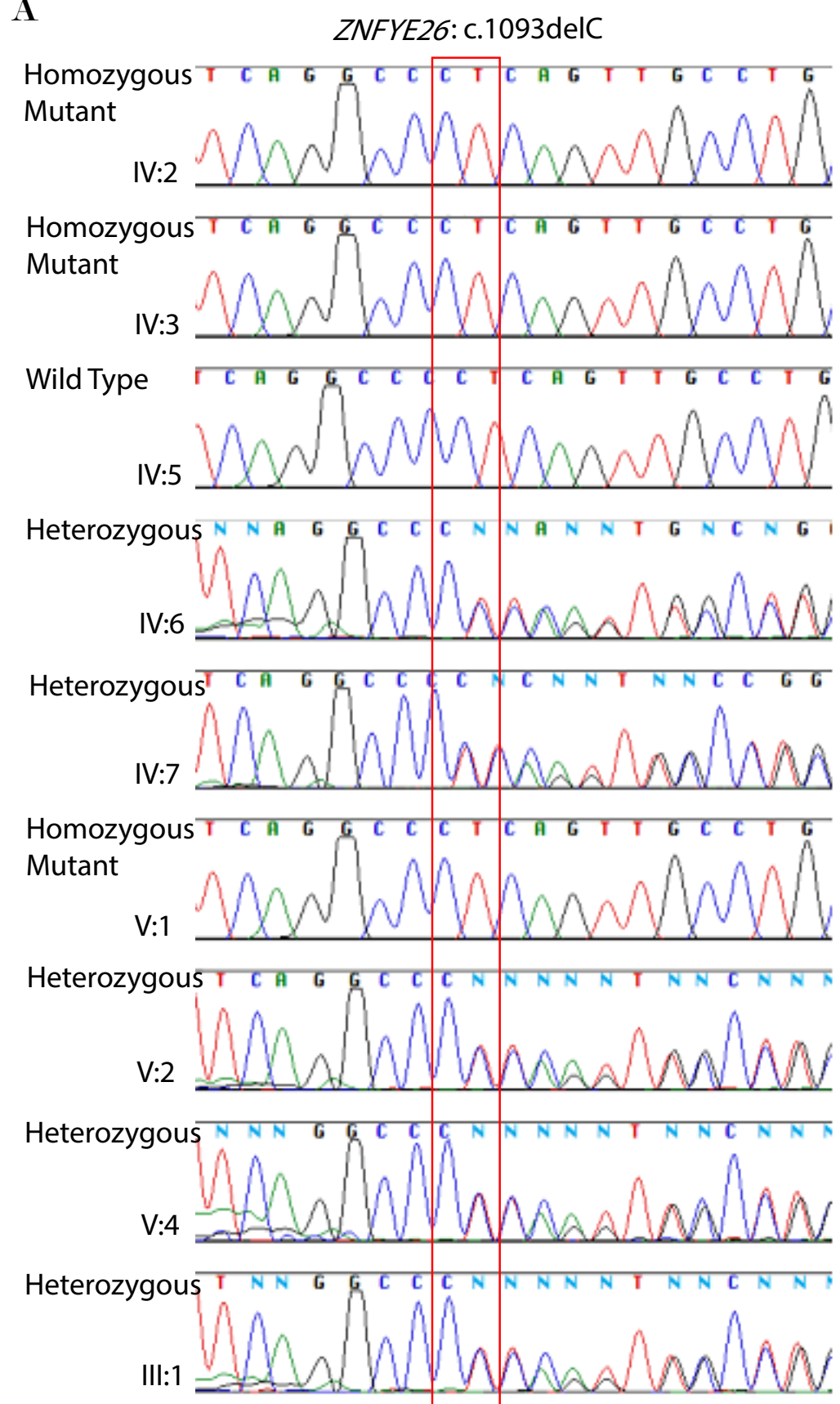**B**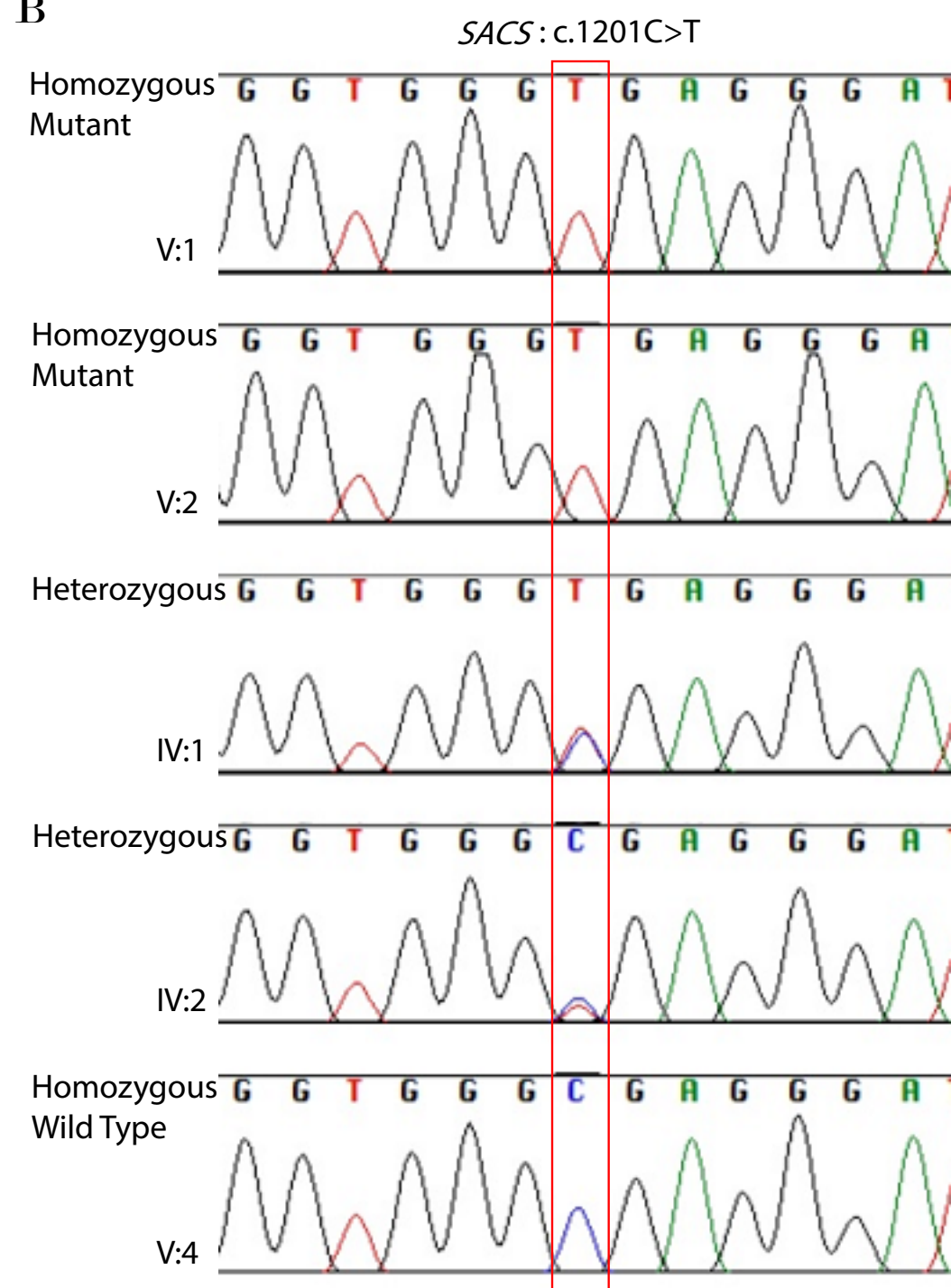**C**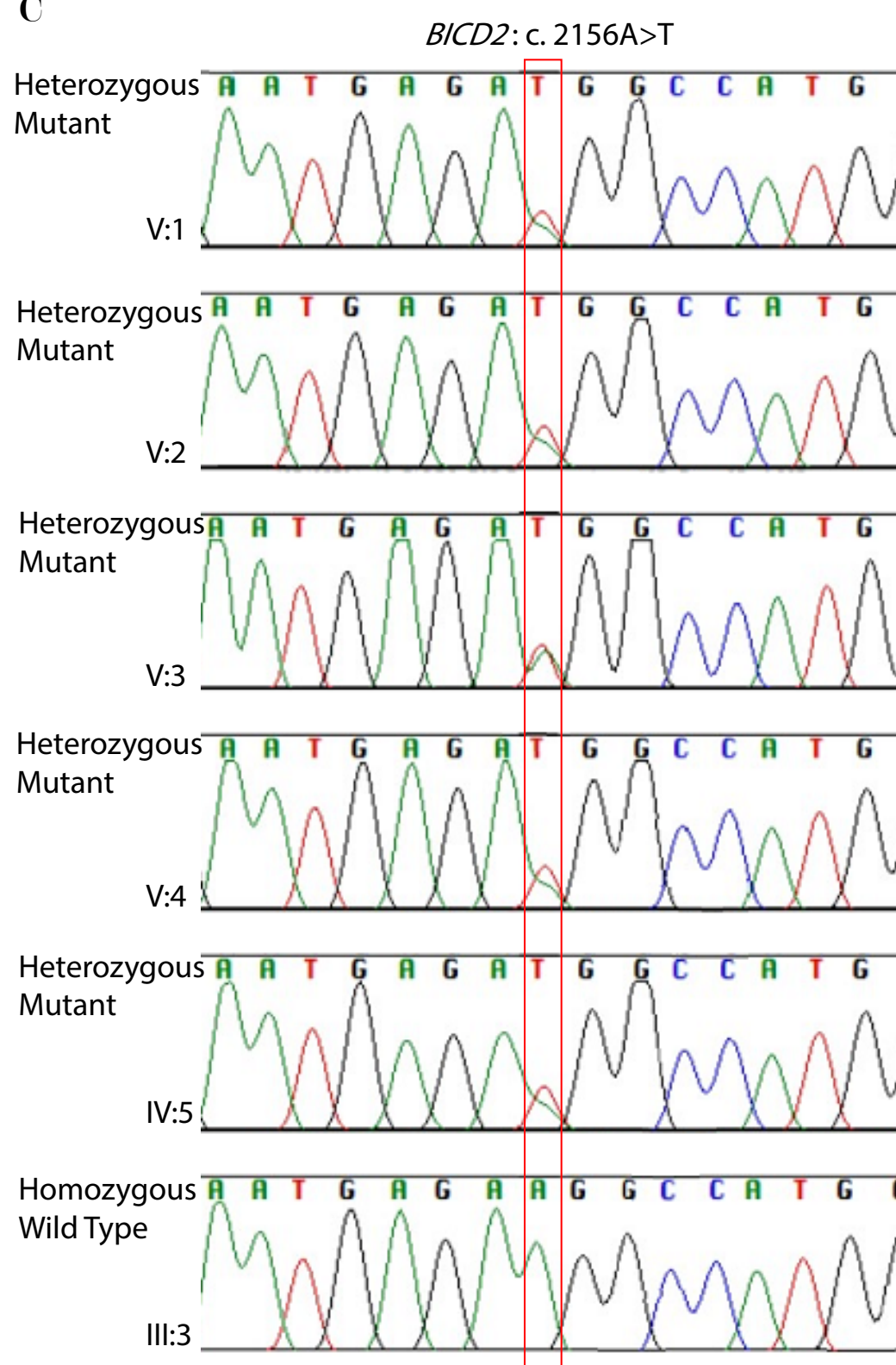**D**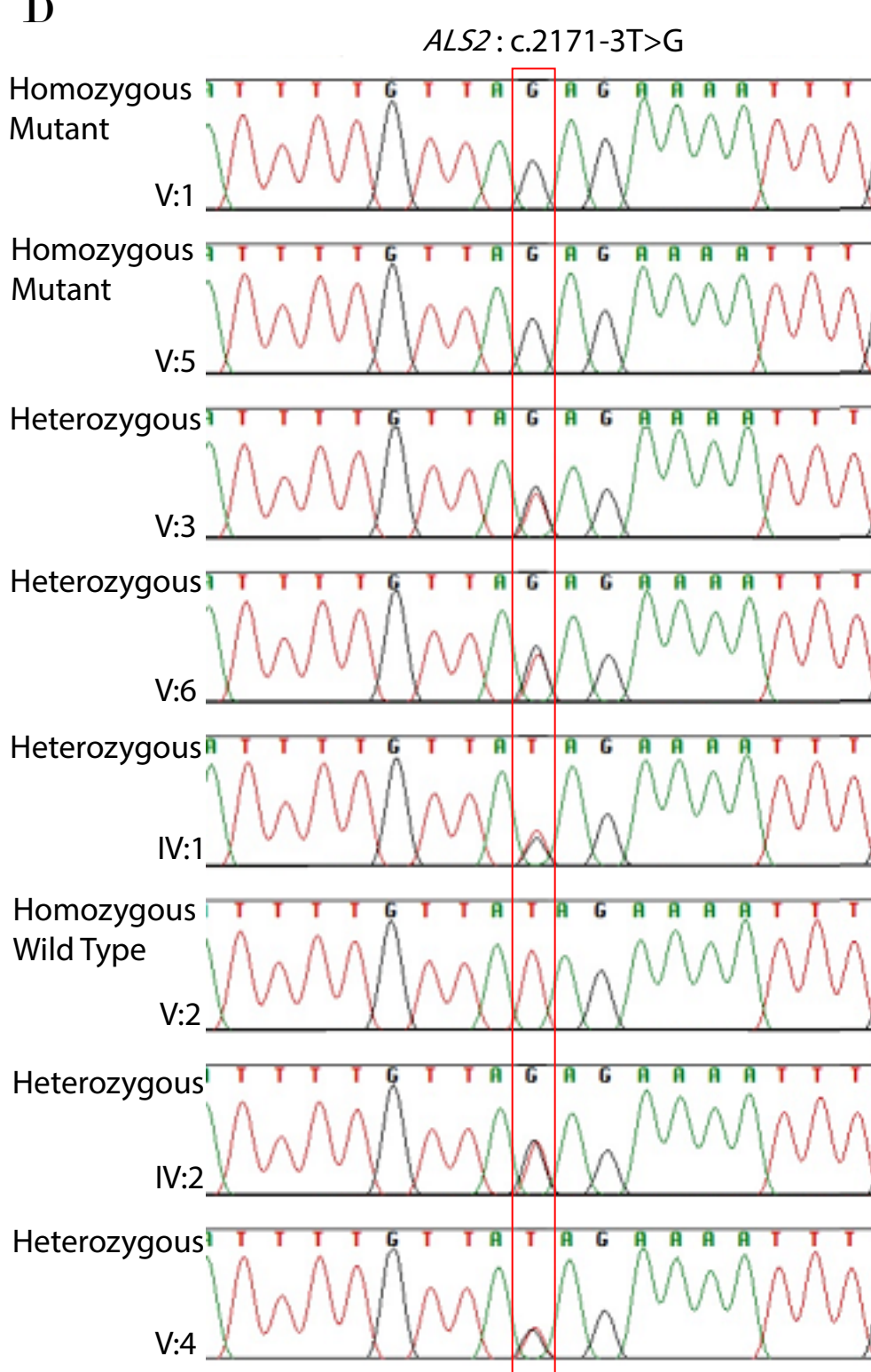**E**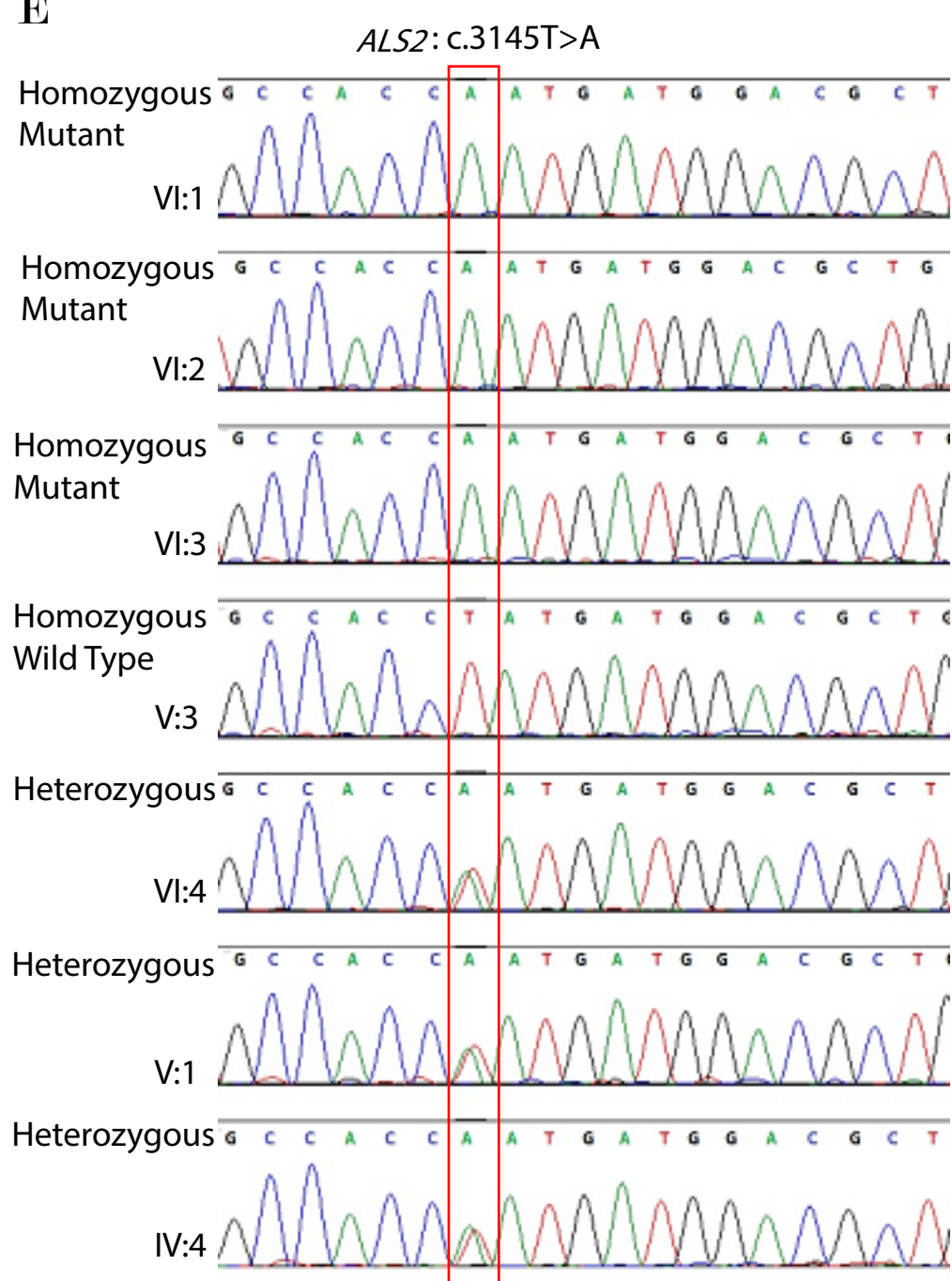**F**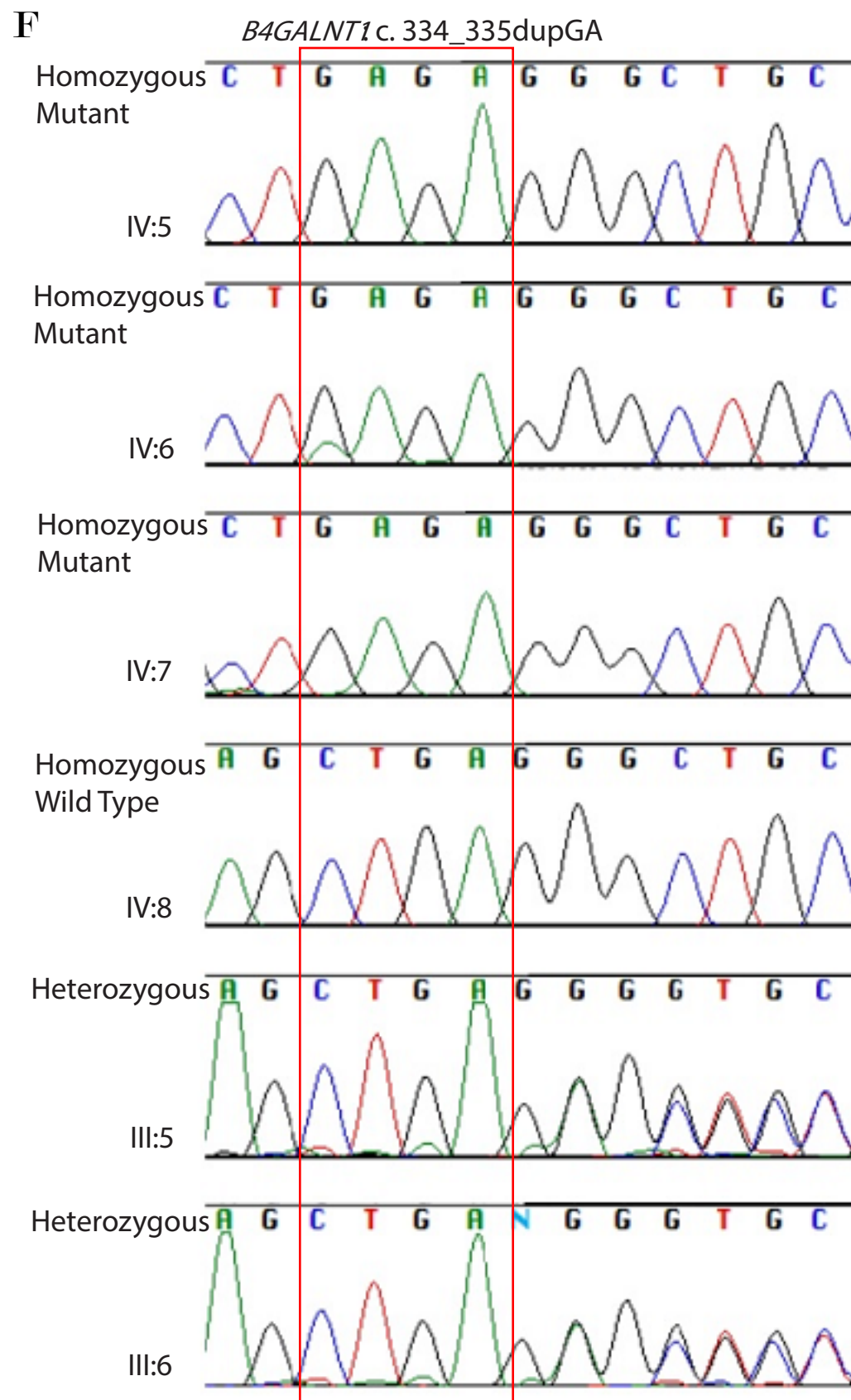**G**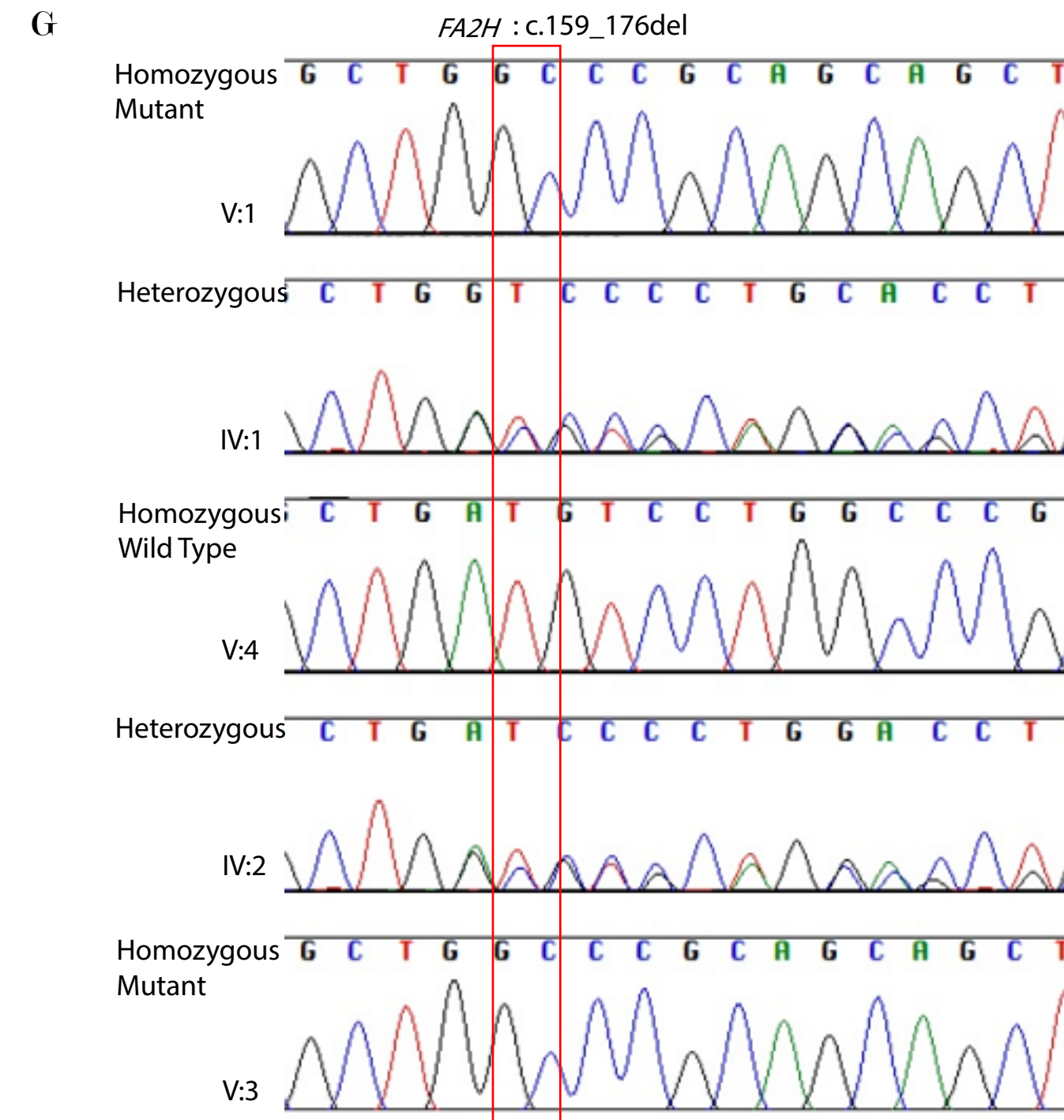**H**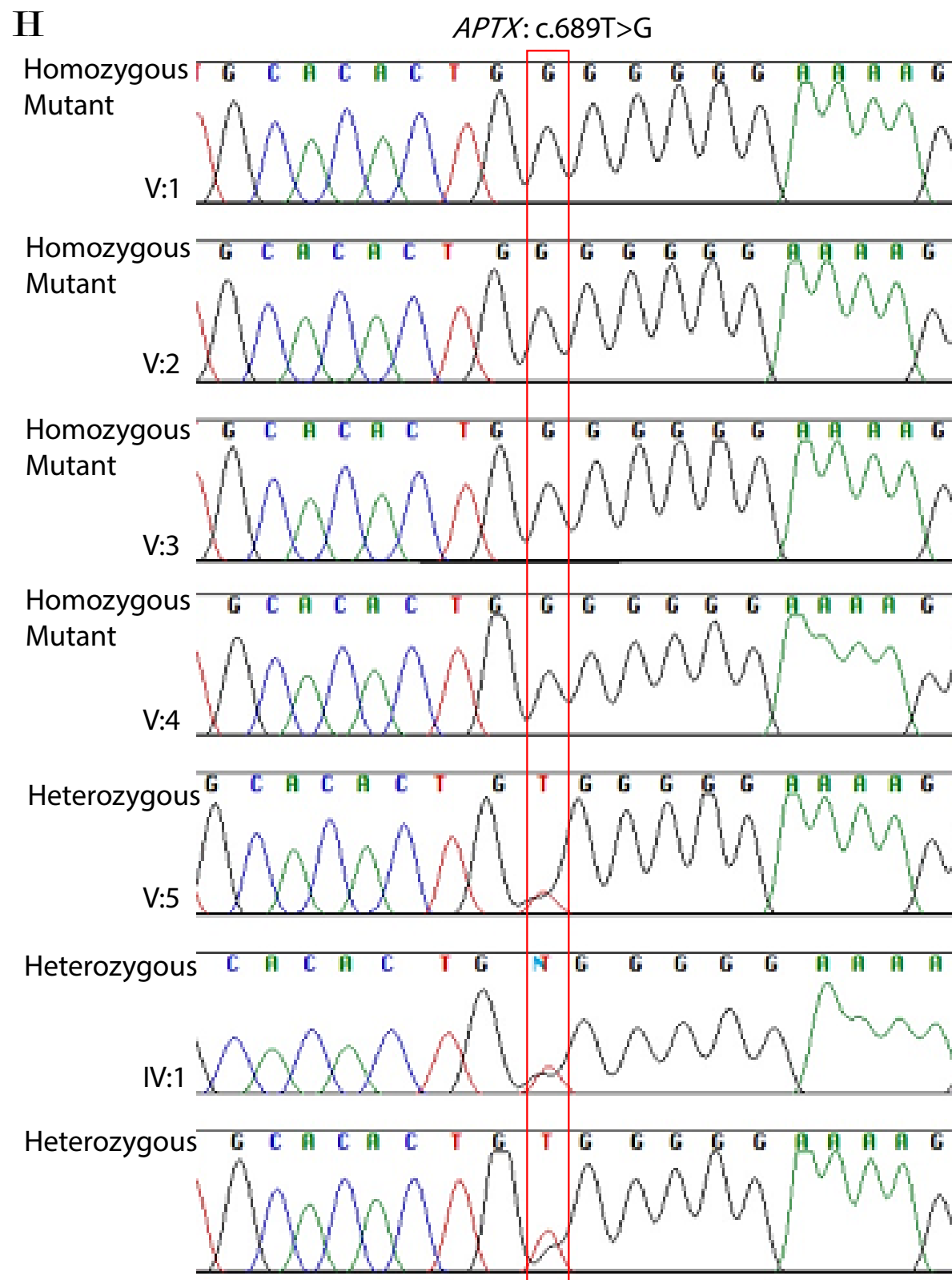**I**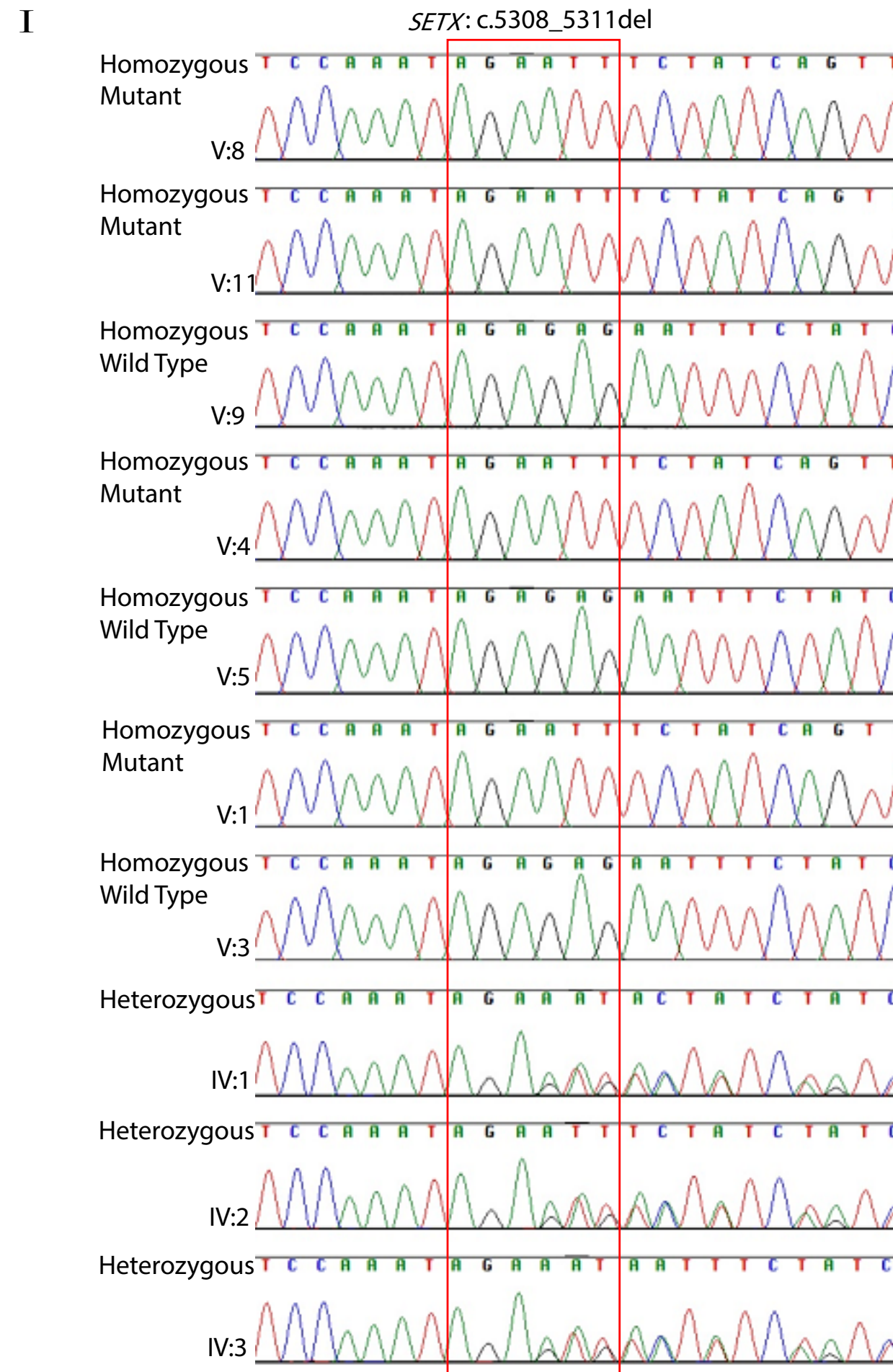

Supplement: Supplementary file 1 [file genes-14-01404-s001.zip › Figure S3.pdf]
